# Resources and Biological Activities of Natural Polyphenols

**DOI:** 10.3390/nu6126020

**Published:** 2014-12-22

**Authors:** An-Na Li, Sha Li, Yu-Jie Zhang, Xiang-Rong Xu, Yu-Ming Chen, Hua-Bin Li

**Affiliations:** 1 Guangdong Provincial Key Laboratory of Food, Nutrition and Health, School of Public Health, Sun Yat-Sen University, Guangzhou 510080, China; E-Mails: lianna28@163.com (A.-N.L.); lishasl0308@163.com (S.L.); zhyujie3@mail2.sysu.edu.cn (Y.-J.Z.); chenyum@mail.sysu.edu.cn (Y.-M.C.); 2 Key Laboratory of Tropical Marine Bio-Resources and Ecology, South China Sea Institute of Oceanology, Chinese Academy of Sciences, Guangzhou 510301, China; E-Mail: xuxr@scsio.ac.cn

**Keywords:** polyphenols, antioxidant, cardioprotection, anticancer, anti-inflammation, antimicrobial, bioavailability

## Abstract

The oxidative stress imposed by reactive oxygen species (ROS) plays an important role in many chronic and degenerative diseases. As an important category of phytochemicals, phenolic compounds universally exist in plants, and have been considered to have high antioxidant ability and free radical scavenging capacity, with the mechanism of inhibiting the enzymes responsible for ROS production and reducing highly oxidized ROS. Therefore, phenolic compounds have attracted increasing attention as potential agents for preventing and treating many oxidative stress-related diseases, such as cardiovascular diseases, cancer, ageing, diabetes mellitus and neurodegenerative diseases. This review summarizes current knowledge of natural polyphenols, including resource, bioactivities, bioavailability and potential toxicity.

## 1. Introduction

The reactive oxygen species (ROS) play an important role in many diseases, such as cardiovascular diseases, cancer, ageing, neurodegenerative diseases and diabetes. Natural polyphenols are the biggest group of phytochemicals, and have attracted more and more attention as potential agents for prevention and treatment of oxidative stress-related diseases.

Natural polyphenols are secondary metabolites of plants, and many of them have been found in plant-based foods. Polyphenols have effects on the bitterness, astringency, color, flavor, odor and oxidative stability in food [1]. Natural polyphenols have been widely studied, and found to possess many important bioactivities [2]. This review summarized current knowledge of natural polyphenols, including resources, bioactivities, bioavailability and potential toxicity. Special attention was paid to the bioactivities, such as antioxidant, cardioprotective, anticancer, anti-ageing, anti-inflammation and antimicrobial properties.

## 2. Resources of Natural Polyphenols

**Figure 1:**
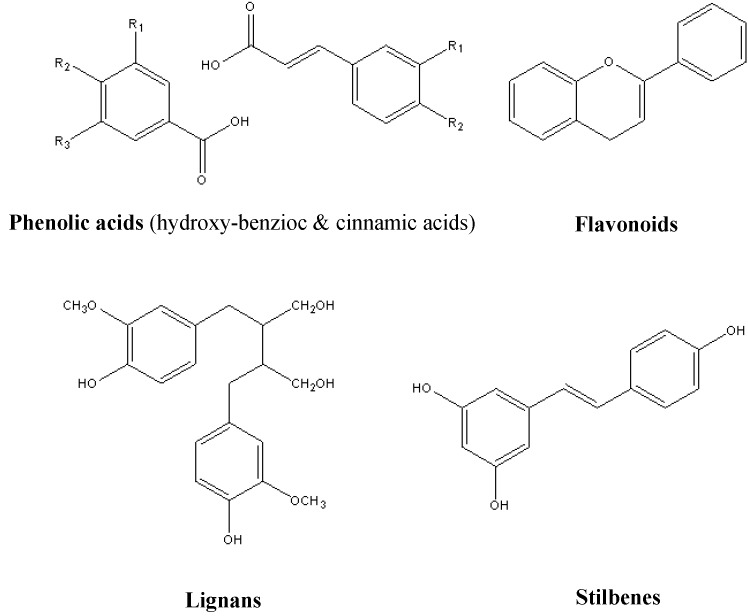
Different groups of polyphenols and their chemical structures.

More than 10,000 polyphenol compounds have been identified in various plants. The typical structural characteristic shared by most polyphenols was built from a common intermediate or a close precursor. Firstly, they occurred in conjugated forms, with one or more sugar residues linked to hydroxyl groups, but direct linkages of the sugar to an aromatic carbon also exist. Linkage with other compounds, such as amines, carboxylic and organic acids, lipids and association with other phenol were also common [3]. Polyphenols could be divided into different groups by the number of phenol rings that they contain and the basis of structural elements that bind these rings, which were classified into several sub-classes, such as the phenolic acids, flavonoids, stilbenes and lignans. Figure 1 shows the different groups of polyphenols and their chemical structures [1,4,5].

Natural polyphenols have been found in many plants and foods, such as fruits, vegetables, tea, cereals, medical plants, microalgae, and edible and wild flowers [6,7,8,9,10,11,12,13,14,15]. Some edible and wild fruits have been evaluated, and found that grape, olive, blueberry, sweetsop, mango and citrus fruits contained high contents of polyphenols [16,17,18,19]. Vegetables are important sources of daily diets and rich in polyphenols. The highest phenolic contents were found in Chinese toon bud, loosestrife, penile leaf, cowpea, caraway, lotus root, sweet potato leaf, soy bean (green), pepper leaf, ginseng leaf, chives, and broccoli [7,20]. In addition, Mediterranean diets are associated with reduced risk of cardiovascular disease due to adequate intake of olive oil and red wine, which contained high contents of polyphenols [21,22]. The pigmented cereals, such as black rice, red rice and purple rice, possessed high total phenolic contents [6]. *Salvia miltiorrhiza* Bge. (danshen), *Polygonum multiflorum* Thunb. (stem) (flowery knotweed), *Rhodiola sacra* Fu (hongjingtian), *Fraxinus rhynchophylla* Hance (ash), and *Prunus persica* (Linn) Batsch. (peach) showed high contents of natural polyphenols among medical plants [23]. *Rosa rugosa* (rose),* Limonium sinuatum* (myosotis),* Pelargonium hortorum* (geranium),* Jatropha integerrima* (peregrina) and *Osmanthus fragrans* (osmanthe) were found to contain lots of natural polyphenols among edible and wild flowers [24]. Furthermore, the contents of polyphenols widely fluctuate between genotype, cultivar, harvest time, and even within organic or conventional [25,26,27].

A number of factors could affect the content of polyphenols in daily food, such as environmental condition, storage, and food processing. For example, sun exposure, rainfall, different types of culture, and the degree of ripeness could affect the concentrations and proportions of polyphenols in different ways [28,29]. Generally, phenolic acid concentrations decreased during ripening, whereas anthocyanins concentrations increased. Inappropriate cooking methods caused significant reduction in the contents of polyphenols. Carrots completely lost their polyphenols after boiling, while steaming and frying had a less negative effect. For broccoli and courgettes, boiling and frying caused a higher loss of polyphenols than steaming [30,31]. However, the evidence was emerging that bioavailability of many protective compounds was enhanced when the vegetables are cooked [32]. Storage also affected the content of polyphenols. After 11 months of storage, the content of phenolic acids decreased 5% to 21% in apple juices [33]. A decrease in the content of free p-coumaric acid was also observed in frozen red raspberries [34]. Another study showed that the antioxidant activities in extra virgin olive oil would decrease after eight months of storage in closed bottles in the dark, which were from loss of phenolic compounds [35]. On the other hand, because of the hydrolysis of complex phenols, an increase in hydroxytyrosol and tyrosol contents during storage was reported [36].

## 3. Bioactivities of Natural Polyphenols

Natural polyphenols have shown numerous biological activities and health benefits for prevention and treatment of age-related diseases, cancers, heart diseases, *etc*. [37].

### 3.1. Antioxidant Activity

Among the notable bioactivities of phenolic compounds, the antioxidant activities have been widely studied, including scavenging of free radicals, inhibition of lipid oxidation, reduction of hydroperoxide formation, and so on [38].

There were many experiments *in vitro* that proved that phenolic compounds were usually major contributors of antioxidant capacities of plants. Rosmarinic acid, ferulic, caffeic, chlorogenic, vanillic, p-hydroxybenzoic acid, p-coumaric acid, protocatechuic acid, and so on were identified to contribute to the antioxidant potential of *Lycopus lucidus* and tea by using DPPH and NO scavenging assays [39,40].

Polyphenols may also function as antioxidants through their effects on plasma, membranes, transcription factors and enzyme activities* in vivo*. Both short-term and long-term effects of a phenolic-rich extract from grape on the antioxidant status of a group of healthy human subjects were investigated [41,42]. In short-term study, 12 fasting subjects (6 men and 6 women) consumed a single dose (400 mL) of the extract. The results indicated that polyphenols from the extract were bioavailable and able to bind with the lipid fraction of serum, therefore reducing lipid peroxidation [41]. In long-term study consecutive 12 week administration of tablets containing 0, 200 or 400 mg grape seed extract (calculated as proanthocyanidin) to 61 healthy subjects with LDL cholesterol levels of 1.00–1.80 mg/mL, effects of such treatment compared to administration of placebo tablets on malondialdehyde-modified LDL were investigated by a single blind method. After 12 weeks, malondialdehyde-modified LDL level in the 200 mg group was significantly reduced compared to the basal level. In the 400 mg group, significant decreases in malondialdehyde-modified LDL level compared to the basal level were seen 6 weeks after the start of administration; and after 6 weeks, significantly lower values were observed compared to those who took placebo tablets. These results suggested that tablets containing grape seed extract exerted reducing effects on oxidized LDL [42]. The antioxidant effects of olive polyphenols on LDL oxidation could be observed after a dietary intake of about 10 mg per day. The 50% of the phenolic compounds contained in olives and virgin olive oil are hydroxytyrosol and derivatives thereof, which could be well absorbed in humans. The human intervention studies showed that olive polyphenols decreased the levels of oxidized-LDL in plasma and positively affected several biomarkers of oxidative damage [43]. Green tea contained abundant of polyphenols, such as catechin, epicatechin and gallate. In a randomized cross-over study, 15 healthy volunteers consumed 500 mL of green tea with different solid contents (1.4, 1.6, 1.8 and 2.0 g/L) to induce a dose-response effect on plasma antioxidant capacity. Ingestion of green tea with solid contents 2.0 g/L significantly increased plasma reducing power at 1 h, 2 h and 4 h. The 1.8 g/L group showed statistical significance at 1 h and 2 h, whereas green tea with solid contents 1.6 g/L was effective only at 1 h and the 1.4 g/L group did not induce any change. This result proved the antioxidant activity of green tea and provided an evidence for the linear correlation between green tea antioxidant content and the extent of the antioxidant effect *in vivo* [44]. In another study, nuts could influence the antioxidant capacity of plasma. Torabian *et al.* assessed the immediate effect of a polyphenols-rich meal (75% of energy from nuts: walnuts or almonds) and a polyphenol-free meal in healthy volunteers [45]. There was a significant increase in plasma polyphenols concentration following both nut meals. The peak concentrations were achieved at 90 min after ingestion of the nut meals. A gradual significant reduction in the susceptibility of plasma to lipid peroxidation was observed after the peak concentration was achieved. The plasma total antioxidant capacity reached its highest point at 150 min post consumption of the nut meals. No changes were observed following consumption of control meal. Consumption of a polyphenol-rich meal, which provided either walnuts or almonds, increased plasma polyphenol concentrations, increased the total antioxidant capacity and reduced plasma lipid peroxidation [45]. Natural polyphenols may also function as antioxidants through their effects on membranes. The antioxidant activity of rosemary extracts is mainly due to phenolic abietane diterpenes and phenolic acids such ascarnosic acid, camosol, rosmadial, genkwanin, and rosmarinic acid. Rosmarinic acid, carnosic acid, and carnosol exhibited similar antioxidant activities in a phospholipid membrane-free assay [46]. Except for protecting the membranes, inhibition of copper-mediated DNA damage has been determined for several polyphenols compounds, such as epicatechin, protocatechuic acid, *n*-propyl gallate, vanillic acid, quercetin, myricetin, (−)-epicatechin-3-gallate, (−)-epigallocatechin, (−)-epigallocatechin-3-gallate and gallic acid [47]. In addition, anthocyanins are thought as major functional components of pigmented rice to provide the bioactivities including antioxidant and free radical scavenging, antitumor, antiatherosclerosis, hypoglycemic, and antiallergic activities [6]. Furthermore, the working mechanism of natural polyphenolic antioxidants has been summarized in two papers [48,49], that is, H atom transfer, electron transfer and metal ions chelation action. Many polyphenols scavenge free radicals through the hydrogen atom transfer mechanism because higher energies are involved in the single electron transfer process. Polyphenols could chelate transition metals through their multiple OH groups and the carbonyl moiety, when present [48].

Polyphenols could be used not only as antioxidant for human but also food preservatives in food industry. Ethanol extracts of Chardonnay grape and black raspberry seed were evaluated for their capacities to suppress lipid oxidation, preserve important fatty acids, and inhibit microbial growth. Both tested seed extracts suppressed lipid oxidation and rancidity development in fish oil. Black raspberry seed extract significantly reduced the degradation of biologically important *n*-3-polyunsaturated fatty acids under accelerated oxidative conditions [50]. Polyphenols from pericarp tissue of postharvest litchi fruit, which were identified as (−)-epicatechin and procyanidin, tended to decrease with increasing skin browning index of litchi fruit during storage at 25 °C [51]. These studies suggested the potential for developing natural food preservatives from rich polyphenols plants to improving food stability, quality, safety, and consumer acceptance.

The antioxidant activities of the extracts from plants have been widely studied in different biological or food system. Antioxidant activities of some extracts/compounds from plants are given in Table 1.

**Table 1:** Antioxidant activities of some extracts/compounds from plants.

Polyphenols	Antioxidant Activity	References
phenolic-rich extract of grape	reduced oxidative stress in serum	[41]
grape seed extract	decreased the oxidated LDL in plasma	[42]
ethanol extracts of Chardonnay grape	food preservatives for fish flesh and oil	[50]
(−)-epicatechin and procyanidin	food preservatives for fruit	[51]
hydroxytyrosol and its derivatives	decreased the oxidized-LDL in plasma and affected several biomarkers of oxidative damage	[43]
catechin, epicatechin and gallate	induced a dose-response effect on plasma antioxidant activity	[44]
anthocyanins	scavenged free radical	[6]
extracts of walnuts and almonds	reduced plasma lipid peroxidation	[45]
ascarnosic acid, camosol, rosmadial, genkwanin, and rosmarinic acid	protected the membranes against oxidative damage	[46]

### 3.2. Cardioprotection Activity

Postprandial hyperlipemia and oxidative stress, a well-defined risk factor for atherosclerosis, could be reduced by polyphenols. Atherosclerosis develops in lesion-prone regions of medium-sized arteries. Atherosclerosis might be present or clinically-silent for decades before becoming active and producing pathological conditions such as acute myocardial infarction, unstable angina, and sudden cardiac death [52]. The rate of myocardial infarction is continually increasing. Numerous studies have shown that dietary polyphenols could reduce the risk of thrombosis, which is one of the leading causes for myocardial infarction, ischemic heart disease, *etc*. [53,54,55]. In addition, epidemiologic studies provided strong evidence that the incidence of cardiovascular disease was low in the Mediterranean area. The consumption of Mediterranean diet which enriched in green vegetables, fruits, fish, and grape wine, particularly red wine, imparted a greater benefit in the prevention of coronary heart disease than the consumption of other diets [56,57].

Both epidemiological and experimental studies suggested that the mild-to-moderate consumption of wine, particularly of red wine, reduced the incidence of mortality and morbidity from coronary heart disease [58,59]. The cardioprotective effects of red wine were attributed to its high content of antioxidant polyphenols including resveratrol and proanthocyanidins. Resveratrol is a polyphenols abundantly found in grapes and wine [60]. Many experiments have been carried out to prove the antioxidant activity of resveratrol *in vitro* and *in vivo*. Mazzone *et al.* investigated two main working mechanisms of five new potential antioxidant analogues of *cis*-resveratrol, H atom and single-electron transfer. The results indicated that the number of hydroxyl groups and the presence of the catechol moiety were the most significant features in determining the order of radical scavenging potentiality [61]. Another systematic study of the antioxidant activity of trans-resveratrol toward hydroxyl and hydroperoxyl radicals was carried out. Because of reacting with hydroxyl at a diffusion-controlled rate, *trans*-resveratrol is thought as an excellent but unselective hydroxyl scavenger that provides antioxidant protection to cells. Reactions between *trans*-resveratrol and the hydroperoxyl radical occur only by phenolic hydrogen abstraction. The total rate coefficient is much smaller than the ones for reactions of trans-resveratrol with hydroxyl, but still important [62].

In a study about human umbilical vein endothelial cells and human umbilical vein endothelial cells-derived EA.hy 9226 cells, resveratrol increased eNOS protein expression and eNOS-derived NO production. In others words, the stimulation of eNOS expression and activity that contributed to the cardiovascular protective effects could be attributed to resveratrol [63]. Furthermore, Thuc *et al.* investigated resveratrol exerted protective effects against cardiac ischaemia/reperfusion and explored its mechanisms [64]. Treatment with resveratrol 15 min before ischemia considerably improved left ventricular functional recovery and infarct size in rat isolated perfused hearts. In cultured neonatal rat cardiomyocytes, resveratrol significantly attenuated the increase in reactive oxygen species and loss of mitochondrial inner membrane potential. Moreover resveratrol suppressed the increase concentrations of Na^+^ and Ca^2+^ in cardiomyocytes after exposure to H_2_O_2_. Resveratrol inhibited the H_2_O_2_-induced translocation of PKC-a, which was believed to activate Na^+^-H^+^ exchanger. The PKC-a-dependent inhibition of Na^+^-H^+^ exchanger and subsequent attenuation of Ca^2+^ overload might provide cardioprotective. Thuc *et al.* also demonstrated that acid-induced activation of the Na^+^-H^+^ exchanger was prevented by resveratrol through measuring changes in intracellular pH recovery after acidification. In addition, sirtuins (SIRTs 1–7) have attracted much attention since the discovery that SIRT1 may be activated by resveratrol. In mammals, regulation of SIRT1 functioned by resveratrol was confirmed in many tissues, including myocardium [65,66]. SIRT1 was known to regulate the activity of several important transcription factors and coactivators that are essential for the protection of numerous types of cardiovascular disturbances, such as Forkhead box O, NO production, Hif-2α [67,68,69]. Resveratrol also had effect on SIRT1-mediated deacetylation which inhibited several potentially detrimental mechanisms associated with myocardial infarction [70,71].

In myocardial ischemic-reperfusion injury experiment, the rats were gavaged with white wine and divided into four groups: control sham, wine-treated sham, control ischemia/reperfusion, and wine-ischemia/reperfusion. After 24 h of reperfusion, significant reduction in infarct size, cardiomyocyte, and endothelial cell apoptosis was observed in wine-ischemia/reperfusion as compared with control ischemia/reperfusion. Echocardiography proved significant increased fractional shortening and ejection fraction following 30 days of reperfusion in wine-ischemia/reperfusion rats. Increased phosphorylation of AKT, Foxo3a, and eNOS were found in wine-treated sham and wine-ischemia/reperfusion, when compared to their respective controls. Significant upregulation of DNA binding activity of NF-κB in the white wine-treated groups was demonstrated by the gel-shift analysis [72]. Furthermore, Samuel et al. have shown that white wine is equally as cardioprotective as red wine, because it contains other compounds such as hydroxycinnamic acids (caffeic acid) and monophenols (tyrosol) [73]. Reduced infarct size and cardiomyocyte apoptosis were observed along with improvement in the myocardial functional parameters as assessed by echocardiography in the tyrosol-treated animals when compared to the non-treated controls. These two constituents of white wine also activated SIRT1 in rat myocardium.

### 3.3. Anticancer Activity

Cancer is a major cause of death across the world. Polyphenols could play an important role in anticancer. The anticancer effects of polyphenols have been observed at mouth, stomach, duodenum, colon, liver, lung, mammary gland or skin. Many polyphenols, such as proanthocyanidins, flavonoid, resveratrol, tannins, epigallocatechin-3-gallate, gallic acid and anthocyanin, have been tested; all of them showed protective effects in some models although their mechanisms of action were found to be different [74].

Natural polyphenols might have a better protective effect on metastatic breast cancer. Scientists therefore investigated the effects of proanthocyanidins on a highly metastatic mouse mammary carcinoma cell line. *In vitro* treatment of breast cancer cells 4T1 resulted in significant inhibition of cellular proliferation and viability, and induction of apoptosis in 4T1 cells in a time- and dose-dependent manner after exposed with proanthocyanidins. The effects of dietary proanthocyanidins were then examined by an *in vivo* model in which 4T1 cells were implanted subcutaneously in Balb/c mice. Proanthocyanidins significantly inhibited the growth of the implanted 4T1 tumor cells. Moreover, the metastasis of tumor cells to the lungs was inhibited significantly and the survival of the mice was enhanced. All these data demonstrated that proanthocyanidins possess chemotherapeutic efficacy against breast cancer including inhibition of metastasis [75]. Berries are rich in anthocyanins, which possess a broad spectrum of therapeutic and anti-carcinogenic properties. Six berry extracts were studied for anti-angiogenic properties. Anti-angiogenic compounds could reduce unwanted growth of blood vessels, which can lead to varicose veins and tumor formation. Berry extracts significantly inhibited both H_2_O_2_- and TNF-α-induced vascular endothelial growth factor expressions, a key regulator of tumor angiogenesis, by human keratinocytes. In an *in vivo* model of angiogenesis, berry extracts significantly inhibited basal MCP-1 and inducible NF-κB transcriptions. Endothelioma cells pretreated with berry extracts showed a diminished ability to form hemangioma and markedly decreased tumor growth by more than 50%. These studies highlight the anti-carcinogenic potential of a novel anthocyanin-rich berry extract [76].

Kuding tea polyphenols presented apoptosis inducing effects of human buccal squamous cell carcinoma cell line BcaCD885 *in vitro* [77]. There were numerous evidences to support the existence of an association between green tea polyphenols consumption and a reduced cancer risk. Prostate cancer is one of the most frequently diagnosed male neoplasia in the Western countries. Recently, an exploratory meta-analysis of observational studies supported the hypothesis that green tea may have a protective effect against prostate cancer. In a total of six epidemiological studies, including two case-control studies and four cohort studies, green tea could reduce developing prostate cancer risk [78]. The most abundant and bioactive components of green tea, catechin, (−)-epigallocatechin-3-gallate and (−)-epigallocatechin gallate, have shown to act as proteasome inhibitors and tumor cell death inducers. In a study, a significant inhibition of breast tumor growth was observed when cultured human breast cancer MDA-MB-231 cells were treated with peracetate-protected (−)-epigallocatechin gallate, associated with increased proteasome inhibition and apoptosis induction in tumor tissues [79]. Gallic acid could also prevent colon carcinogenesis by inhibiting DNA damage. There was an experiment evaluated the efficacy of gallic acid supplementation on tissue lipid peroxidation and antioxidant defense system in 1,2-dimethyhydrazine induced colon carcinogenesis in male Wistar rats. Decreased lipid peroxidation products such as lipid hydroperoxides and conjugated dienes were observed. Gallic acid supplementation could significantly elevate the diminished levels of antioxidants such as SOD, catalase, reduced glutathione, glutathione reductase and glutathione peroxidase in the tissues of 1,2-dimethyhydrazine treated rats. Moreover, enhanced activity of ascorbic acid and α-tocopherol levels were also observed after gallic acid supplementation [80].

Except for gallic acid, flavone could prevent colon carcinogenesis too. Wenzel *et al*. investigated how 2-phenyl-4*H*-1-benzopyran-4-one, the core structure of the flavones, affects HT-29 human colon cancer cells to understand the molecular basis of the putative anticancer activity of flavonoids [81]. Flavone was found to reduce cell proliferation in HT-29 cells and to potently induce differentiation as well as apoptosis. The flavonoid proved to be a stronger apoptosis inducer than the clinically established antitumor agent camptothecin. Moreover, flavone, but not camptothecin, displayed a high selectivity for the induction of apoptosis and of growth inhibition only in the transformed colonocytes. The results showed that flavone induced effectively programmed cell death, differentiation, and growth inhibition in transformed colonocytes by acting at the mRNA levels of genes involved in these processes [81]. Flavone might be a potent new cytostatic compound with improved selectivity toward transformed cells because these genes play a crucial role in colon carcinogenesis.

Resveratrol could inhibit each stage of multistage carcinogenesis, scavenge incipient populations of androgen-dependent prostate cancer cells through androgen receptor antagonism, and scavenge incipient populations of androgen-independent prostate cancer cells by short-circuiting the epidermal growth factor-receptor-dependent autocrine loops in the cancer cells. Resveratrol is a prototype of a plethora of bioactive polyphenols in the food supply that has just begun to be mined for cancer preventive agents [82]. The identification of resveratrol as a cancer preventive agent is largely owed to its high abundance in nature. For example, polyphenolic grape seed fractions were shown recently to potently antagonize chemical carcinogenesis, and resveratrol accounts for 5%–10% of the grape skin biomass. Thus, resveratrol may represent the tip of the iceberg of a broad class of stilbene and related polyphenolic natural products for cancer prevention [82]. In addition, in a survey of bioactive ellagitannins with a macrocyclic structure and/or a gluconic acid core, some new oligomeric ellagitannins were found in species of *Myrtaceae* and *Elaeagnaceae*. Both cytotoxic activity against human oral tumor cell lines and antibacterial activity against *Helicobacter pylori* were evaluated for the ellagitannins obtained from both plants. The macrocyclic dimers showed a remarkable cytotoxicity against human oral squamous cell carcinoma, but had no effect on normal cells. These results demonstrated active tannins induced apoptosis of tumor cells [83,84].

Polyphenols influence the metabolism of pro-carcinogens by modulating the expression of cytochrome P450 enzymes involved in their activation to carcinogens [85]. Polyphenols could also protect genomic DNA by mobilizing of endogenous copper possibly chromatin bound copper [86]. Many mechanisms of action have been demonstrated for chemopreventive effect of polyphenols, such as antiproliferation, antioxidation, estrogenic/antiestrogenic activity, induction of cell cycle arrest or apoptosis, induction of detoxification enzymes, regulation of the host immune system and changes in cellular signaling [1].

The anticancer activities of some phenolic compounds have been studied widely, and the results are summarized in Table 2.

**Table 2:** Anticancer activities of some polyphenols.

Polyphenols	Effects	References
proanthocyanidins	inhibited breast cancer metastasis	[75]
anthocyanin	repaired/protected genomic DNA integrity and retarded blood vessel growth in some tumors	[76]
epigallocatechin-3-gallate	inhibited proteasome and induced tumor cell death; prevented prostate cancer	[78,79]
gallic acid	prevented colon carcinogenesis by inhibiting DNA damage	[80]
2-phenyl-4*H*-1-benzopyran-4-one	induced apoptosis	[81]
resveratrol	inhibited each stage of multistage arcinogenesis; scavenged incipient populations of androgen-dependent/independent prostate cancer cells	[82]
ellagitannins	induced apoptosis of human oral tumor cell lines	[83]

### 3.4. Anti-Inflammation Activity

Excessive inflammation is considered as a critical factor in many human diseases, including obesity, type II diabetes, cardiovascular diseases, neurodegenerative diseases and ageing. Polyphenols have shown significant anti-inflammation effects *in vivo* and *in vitro* [87].

There was a study about the potential anti-inflammatory effect of 18 polyphenol metabolites which derived from colon microbiota [88]. They were screened by measuring prostaglandin E-2 production; hydrocaffeic, dihydroxyphenyl acetic, and hydroferulic acid could inhibit more than 50% prostaglandin E-2 production. Subsequently, these three compounds were tested with the writhing and paw pressure test in rodents and all the compounds showed an anti-inflammatory effect. The effect of hydrocaffeic administered orally (50 mg/kg) was also tested in the dextran sodium sulfate induced colitis model. Weight loss and fecal water content were more pronounced in dextran sodium sulfate rats than in dextran sodium sulfate-hydrocaffeic treated rats. Hydrocaffeic treatment could also diminish the expression of the cytokines IL-1β, IL-8, and TNF-α, reduced MDA levels and oxidative DNA damage in distal colon mucosa [88].

Martinez-Dominguez *et al.* demonstrated that virgin olive oil with a higher content of polyphenols showed protective effects in inflammation [89]. In carrageenan oedema test, the inflammation indices of animals fed on a diet rich in olive oil were lower compared to animals fed with oils high in oleic acid and polyunsaturated fatty acids. In established adjuvant arthritis, the 15% virgin olive oil supplemented with 600 ppm polyphenols from this oil diet was even more effective than 15% fish oil diet in the prevention of inflammation. Both groups administered to Indomethacin, and a strong inhibitory effect throughout the inflammatory process was observed in the group of 15% virgin olive oil supplemented with 600 ppm polyphenols. Another study investigated the effects of polyphenols found in extra virgin olive oil on inflammatory mediator production by human mononuclear cells. Diluted human blood cultures were stimulated with lipopolysaccharide in the presence of polyphenols at concentrations of 10^−7^ to 10^−4^ M. At a concentration of 10^−4^ M, oleuropein glycoside inhibited interleukin 1β production by 80%, whereas caffeic acid inhibited production by 40%. Kaempferol decreased the concentration of prostaglandin E-2 production by 95% at a concentration of 10^−4^ M [90].

Consumption of green tea polyphenols resulted in reduction in the levels of markers of inflammation and proinflammatory cytokines in chronically UVB-exposed skin and skin tumors of wild-type mice [91]. Catechins are abundant in green tea. Their anti-inflammatory effects are activated through a variety of different mechanisms, including modulation of nitric oxide synthase isoforms. Catechins’ actions of attenuating the inflammatory response may account for their confirmed neuroprotective capabilities following cerebral ischemia [92]. Polyphenols extracted from *Hibiscus sabdariffa* L. was found to have an anti-inflammatory potency both *in vitro* and *in vivo*. Polyphenols extracted from *Hibiscus sabdariffa* L. were proved to have anti-inflammatory effects on nitrite and prostaglandin E-2 in lipopolysaccharide treated RAW264.7 cells. Sequentially, an animal model of lipopolysaccharide-induced hepatic inflammation was performed. Polyphenols significantly decreased the serum levels of alanine and aspartate aminotransferase in lipopolysaccharide-treated rats. In the liver of rats, lipid peroxidation and liver lesions decreased, and catalase activity and glutathione increased [93]. Polyphenols extract from the *Tunisian quince* was reported as a non-toxic, cost-effective natural agent of anti-inflammatory. Quince peel polyphenols extract could inhibit human macrophages secrete pro-inflammatory cytokine TNF-α and the chemokine IL-8 in a dose-dependent manner. Concomitantly, quince polyphenols enhanced the level of the anti-inflammatory cytokine IL-1β secreted by lipopolysaccharide-treated macrophages. Biochemical analysis showed that quince polyphenols extract inhibited the lipopolysaccharide-mediated activation of three major cellular pro-inflammatory effectors, NF-κB, p38MAPK and AKT. This study indicated that quince peel polyphenols extract induced a potent anti-inflammatory effect [94]. Quince peel contains a lot of plant flavonoids. There have been numerous topical applications of plant extracts having flavonoids known as anti-inflammatory compounds [95]. Quercetin, a kind of flavonoids, could play a critical role in reducing the risk of atherosclerosis through activation of inflammatory signaling [96]. Consumption of polyphenol-rich foods like grapes or their by-products, which have anti-inflammatory properties, could reduce inflammation. Resveratrol commonly found in grape products has been reported to reduce inflammation in many mechanisms. For example, it could block proinflammatory cytokines, suppress inflammatory gene expression and activate transcription factors that antagonize chronic inflammation. Thus, polyphenol-rich grape products reduce obesity-mediated chronic inflammation [17,97].

In recent years, we were allowed to understand interactions of different polyphenols with basic mechanisms of inflammatory response by the modern methods in cell and molecular biology. Polyphenols could affect vascular inflammation and injury not only as antioxidants but also as modulators of inflammatory redox signaling pathways [96,98]. There are several anti-inflammatory mechanisms of polyphenols* in vivo*. One of the important anti-inflammatory mechanisms is the inhibition of eicosanoids generating enzymes including phospholipase A_2_ and cyclooxygenase [99]. Polyphenols were able to modulate cyclooxygenase-2 activity and gene expression in different cell types [95,100]. Nitric oxide (NO) is an essential component in maintain vascular health, and is a key intravascular anti-thrombotic factor, but it provokes inflammatory response if converted to peroxynitrite, in the presence of free radicals. There were experiments suggesting that polyphenols inhibited NO release by suppressing NOS enzymes expression and NOS activity [101]. Cytokines, the major mediators of local and intercellular communications in immune and inflammatory processes, were modulated by polyphenols [90]. NF-κB plays a pivotal role in immune, inflammatory, stress, proliferative and apoptotic responses [102]. The inhibition of NF-κB is generally thought a useful strategy for treatment of inflammatory disorders [103]. Rahman *et al.* suggested that dietary polyphenols could work as modifiers of signal transduction pathways to elicit their beneficial effects [104]. NF-κB required assistance from other sequence specific transcription factors among the MAPK. Polyphenols have been demonstrated to modulate MAPK pathway by acting on several steps of the activation cascade and consequently on downstream effectors [105]. These studies strongly proved the idea that polyphenols had the capacity to modulate the immune response and had a potential anti-inflammatory activity [88].

### 3.5. Antimicrobial Effect

Polyphenols have been demonstrated potential antibacterial, antifungal and antiviral activities [106,107,108]. Alvarez-Suarez *et al.* analyzed several Cuban honeys to determine their total phenolic, flavonoid, ascorbic acid, amino acid, protein and carotenoid contents as well as their antimicrobial capacities. The antimicrobial activity was screened by two Gram-positive and Gram-negative bacteria. *Staphylococcus aureus* was the most sensitive microorganism while *Pseudomonas aeruginos* was the least sensitive microorganism. *Bacillus subtilis* and *Escherichia coli* were both moderately sensitive to honey antimicrobial activity [109]. In another study, antimicrobial effects of the wood-associated polyphenolic compounds were assessed against both Gram-negative (*Salmonella*) and Gram-positive bacteria (*Listeria monocytogenes*). The stilbenes pinosylvin, pinosylvin monomethyl ether and piceatannol were demonstrated to have clear antimicrobial activities. The destabilization of the outer membrane of Gram-negative microorganisms, as well as interactions with the cell membrane might be one of the specific mechanisms behind the antibacterial action. *Listeria monocytogenes* was particularly sensitive to pinosylvin [110]. In addition, polyphenol extracts from industrial sour cherry pomaces were characterized on polyphenol composition and antimicrobial activity. Extracts of pomace contained anthocyanins, hydroxycinnamic acids and flavonoids. The antimicrobial effect of sour cherry polyphenol extracts was tested against *Salmonella*, *Escherichia coli* 0157:H7 and *Listeria* spp. The sour cherry extracts reduced the growth of *Salmonella* and *Escherichia coli* O157:H7 at concentrations higher than 2500 μg/mL, and inhibited *Listeria* spp*.* growth [111]. Furthermore, the polyphenols from tobacco leaf were analyzed by reverse-phase high-performance liquid chromatography and electrospray ionization mass spectrometry. The dominant polyphenols in tobacco leaf were identified as chlorogenic acid and rutin. The proliferation inhibition activities on *Escherichia coli*, *Staphylococcus aureus* and *Bacillus subtilis* were also measured for evaluating the antimicrobial activity of polyphenols from tobacco leaf. The diameters of inhibition zones were 20.23 ± 0.42, 17.66 ± 0.86 and 12.89 ± 0.29 mm, respectively. This result showed that polyphenols from tobacco leaf had great potential as antimicrobial agent [112]. The inhibitory action of tea polyphenols towards the development and growth of bacterial spores was also examined. Tea polyphenols showed antibacterial effects towards *Bacillus stearothemophilus*. The heat resistance of *Bacillus stearothemophilus* spores and *Clostridium thermoaceticum* spores were reduced by the addition of tea polyphenols. (−)-Epigallocatechin gallate, which is the main component of tea polyphenols, showed strong activity against both *Bacillus stearothemophilus* and *Clostridium thermoaceticum*. The heat resistance of these bacterial spores was more rapidly decreased by the addition of tea polyphenols at high temperatures [113]. Epigallocatechin-3-gallate, the major catechin found in green tea, has shown to have antimicrobial effects against a number of bacterial pathogens. The antimicrobial activity against *Stenotrophomonas maltophilia* was proved *in vitro*. The researchers evaluated the *in vitro* activity of this compound against 40 clinical isolates of *Stenotrophomonas maltophilia*. Minimal inhibitory concentrations for 50% and 90% of the organisms were 256 mg/L and 512 mg/L when determined by agar dilution and broth microdilution, respectively. In time-kill assays, the bactericidal activity of epigallocatechin-3-gallate was analyzed by viable colony counts as well as a colorimetric assay. Epigallocatechin-3-gallate was slowly bactericidal at four times the minimal inhibitory concentrations, with a 2.5 log reduction in viable bacteria at 24 h [114].

Polyphenols were able to suppress a number of microbial virulence factors, such as reduction of host ligands adhesion, inhibition of biofilm formation, neutralization of bacterial toxins, and show synergism with antibiotics [115]. Polyphenols were proved to have conjunctive use with antibiotics in order to potentiate their efficacy, to lower antibiotic dose, and therefore to reduce antibiotic adverse reactions. Jayaraman *et al.* investigated the* in vitro* activities of seven antibiotics (ciprofloxacin, ceftazidime, tetracycline, trimethoprim, sulfamethoxazole, polymyxin B and piperacillin) and six polyphenols (protocatechuic acid, gallic acid, ellagic acid, rutin, berberine and myricetin) against five *Pseudomonas aeruginosa* isolates, alone and in combination [116]. All the polyphenols under investigation showed potential inhibitory activity against *Pseudomonas aeruginosa*. The combinations of sulfamethoxazole plus protocatechuic acid, sulfamethoxazole plus ellagic acid, sulfamethoxazole plus gallic acid and tetracycline plus gallic acid showed synergistic mode of interaction. These findings had potential implications in delaying the development of resistance as the antibacterial effect is achieved with lower concentrations of drugs. In another study, antibacterial and antifungal activities of an ample number of phenolic compounds isolated from *Quercus ilex* leaves, including flavonoids, proanthocyanidins, and phenolic acids, were investigated. The isolated compounds were tested for their antimicrobial effects against eight human bacterial species and 14 fungal species. They all showed potential antimicrobial effects. The most potent compound, proanthocyanidins, was tested in combination with the conventional fungicides, bifonazole and ketoconazole, to evaluate possible synergistic effects. When combined with bifonazole and ketoconazole, proanthocyanidins increased the activity of both conventional fungicides [117]. *Chlamydia pneumoniae* is a human pathogen that causes multiple diseases worldwide. The use of the macrolide clarithromycin and the fluoroquinolone ofloxacin has improved the treatment of chlamydial infection, but therapy failure is still a major problem. Rizzo *et al.* studied the pretreatment with natural polyphenols and subsequent treatment with clarithromycin or ofloxacin [118]. Resveratrol and quercetin improved the anti-chlamydial effect of clarithromycin and ofloxacin. Resveratrol at 40 µM and quercetin at 20 µM exhibited significant growth inhibition on *Chlamydia pneumoniae* in presence of clarithromycin or ofloxacin compared to controls. They also demonstrated that both resveratrol and quercetin decreased IL-17 and IL-23 production in *Chlamydia pneumoniae*-infected cells. These results showed the combined treatment may afford a synergistic effect in controlling *Chlamydia infections*. Considering the increase in microbial resistance against traditional antibiotic therapy, polyphenols have been proposed to develop innovative therapies for the treatment of various microbial infections [119]. Ethanol extract of *Turnera ulmifolia* L. was tested for its antimicrobial activity alone or in combination with aminoglycosides against a methicillin-resistant *Staphylococcus aureus* strain. The synergistic effect of this extract on gentamicin and kanamycin was demonstrated. These data suggested that the extract from *Turnera ulmifolia* could be used as a source of plant-derived natural products against the problem of bacterial resistance to antibiotics [120]. In addition, the antibacterial, synergy effects and primary mechanism of action of galangin and ceftazidime against *Staphylococcus aureu* DMST 20651 were investigated. The combination of ceftazidime at 5 μg/mL and 5 μg/mL galangin exhibited synergistic effect by reduced the colony forming units of this strain over 6 and throughout 24 h. Electron-microscopy clearly showed that the combination of galangin and ceftazidime caused damage to the ultrastructures of the cells of this strain. From this experiment, we knew that galangin exhibited the potential to reverse bacterial resistance to β-lactam antibiotics against penicillin-resistant *Staphylococcus aureu*. These findings lead to developing a new generation of phytopharmaceuticals that may use galangin in combination with ceftazidime to treat penicillin-resistant *Staphylococcus aureu* that currently is an almost untreatable microorganism [121].

Despite the use of polyphenols in treatment, the antimicrobial property of polyphenols was also proposed to develop new food antimicrobial and preservatives for increasing consumer pressure on the food industry to avoid synthetic preservatives. In a study, the combinations and the synergistic antibacterial effects of flavonoid and non-flavonoid phenolic compound against *Escherichia coli* ATCC 35218 were investigated. In nutrient medium, the combinations of gallic and protocatechuic acids, gallic and caffeic acids, and rutin and quercetin were the best antibacterial agents, with synergistic effects, and were selected to test their activity in a meat model system. All combinations diminished the bacterial growth, without cellular death at 20 °C. The combinations of gallic and caffeic acids and rutin and quercetin were the most effective at 4 °C. The lowest decimal reduction time was found with the rutin and quercetin combination. These results demonstrated a synergistic effect of the selected combination of flavonoid or non-flavonoid compounds with an important antibacterial effect in meat [122]. In addition, the laboratory tests showed that the minimal inhibitory concentration of the polyphenols present in the oil mill waste water against *Xanthomonas campestris*, the crucifer seed-borne phytopathogen, was equal to 2.5 mg/mL of total polyphenols expressed as caffeic acid. The SDS-PAGE analysis performed with crude extract of *Xanthomonas campestris* confirmed that the phenolic compounds reacted with the protein of the bacterial cells wall disrupting them. The disinfection trials of cauliflower seeds used for greenhouse plant production demonstrated that the polyphenols present in the oil mill waste water could completely control the seed-borne phytopathogen *Xanthomonas campestris* without damaging the germinability of the crucifer seeds and the metallic greenhouse structures. Thus, the polyphenols present in the oil mill waste water could be used to disinfect seeds in greenhouses [123].

### 3.6. Anti-Ageing Effect

Due to notable antioxidant activity of polyphenols, they might be beneficial in reversing the course of neuronal and behavioral ageing. The researchers evaluated the role of grape seed extract on antioxidant status in discrete regions of the central nervous system of young and aged rats [124]. Age-associated increase in lipid peroxidation was observed in the spinal cord, cerebral cortex, striatum and the hippocampus regions of aged rats. Activities of antioxidant enzymes and levels of non-enzymic antioxidants were found to be significantly decreased in all the brain regions studied in aged rats when compared to young rats. However, normalized lipid peroxidation and antioxidant defenses were reported in the grape seed extract-supplemented aged rats. These findings demonstrated that grape seed extract decreased the incidence of free radical-induced lipid peroxidation in the central nervous system of aged rats. Latter, they discovered that polyphenols inhibited the accumulation of age-related oxidative DNA damages in neural tissue. In the following experiments, they evaluated the salubrious role of grape seed extract on accumulation of oxidative DNA damage products such as 8-OHdG and DNA protein cross-links in aged rats. The results revealed that grape seed extract has inhibiting effect on the accumulation of age-related oxidative DNA damages in spinal cord and in various brain regions such as cerebral cortex, striatum and hippocampus [125]. In addition, the risk of Parkinson’s disease, a neurological disorder characterized by degeneration of dopaminergic neurons, could be reduced by polyphenols [1]. The neuroprotective property of green tea extract and (−)-epigallocatechin-3-gallate in mice model of Parkinson’s disease was proven. Parkinson’s disease was caused by dopamine neuron loss in substantia nigra concomitant with depletion in striatal dopamine and tyrosine hydroxylase protein levels. Pretreatment of mice with either green tea extract (0.5 and 1 mg/kg) or (−)-epigallocatechin-3-gallate (2 and 10 mg/kg) prevented these conditions. (−)-Epigallocatechin-3-gallate itself also increased the activities of superoxide dismutase and catalase in the brain [126]. Brain penetrating property of polyphenols, as well as their antioxidant and iron-chelating properties may make such compounds used for prevention and treatment of a variety of neurodegenerative diseases. Rutin could inhibit Aβ aggregation and cytotoxicity, attenuate oxidative stress, and decrease the production of nitric oxide and proinflammatory cytokines *in vitro*. Xu *et al.* investigated the effect of rutin on APPswe/PS1dE9 transgenic mice. The results demonstrated that orally administered rutin significantly attenuated memory deficits in Alzheimer’s disease transgenic mice, decreased oligomeric Aβ level, increased super oxide dismutase activity and glutathione/glutathione disulfide ratio, reduced glutathione disulfide and MDA levels, downregulated microgliosis and astrocytosis, and decreased interleukin IL-1β and IL-6 levels in the brain. These results indicated that rutin was a promising agent for Alzheimer’s disease treatment because of its antioxidant, anti-inflammatory, and reducing Aβ oligomer activities [127]. Because of strong antioxidant and anti-inflammatory properties, maize bran polyphenol and ferulic acid were also reported to be beneficial in Alzheimer’s disease [128].

### 3.7. Other Bioactivities of Polyphenols

Except for the above introduced bioactivities, polyphenols have shown several other health beneficial effects. There were evidences showing that polyphenols had a protective response to skin damage, erythema and lipid peroxidation from UV exposure [91,129]. The protective effects of (−)-epigallocatechin-3-gallate on the ultraviolet-induced skin damage were studied in guinea pigs, hairless mice and human dermal fibroblast cultures. The amount of lipid peroxides produced in the control and (−)-epigallocatechin-3-gallate treated group were 838 ± 144 and 286 ± 57 nmol/mg at 18 h after UV irradiation, respectively. UVB-induced erythema was also hugely reduced in the (−)-epigallocatechin-3-gallate treated group. The erythema relative index of the control and the (−)-epigallocatechin-3-gallate treated group were 311 ± 45 and 191 ± 49 nmol/mg at 16 h after UV irradiation, respectively. (−)-Epigallocatechin-3-gallate reduced the phenomenon of skin damage, such as roughness, sagginess and the decrease of dermal collagen, induced by UVA in hairless mouse skin. (−)-Epigallocatechin-3-gallate treatment could also block the UV-induced increase of collagen secretion and collagenase mRNA level in fibroblast culture [130]. In addition, consumption of green tea polyphenols prevents photocarcinogenesis in mice. Using wild-type mice and an established photocarcinogenesis protocol, Meeran *et al.* found that administration of green tea polyphenols (0.2%, w/v) in drinking water significantly reduced UVB-induced tumor development in wild-type mice. UVB-induced DNA damage was resolved rapidly in green tea polyphenols-treated wild-type mice than untreated wild-type mice [91].

The protection of kidney by polyphenols has also been reported [130]. Polyphenols could protect the kidney against rhabdomyolysis following glycerol injection. This renoprotective effect could be observed much more often in humans with chronic red wine consumption [131]. Except for kidney, polyphenols might protect against obstructive lung disease. A high intake of the soy isoflavone and genistein was associated with better lung function in asthmatic patients. A community-based, cross-sectional study of 1601 young adults, who were initially recruited by random selection from the federal electoral rolls, showed that the consumption of soy beverages was associated with a range of asthma definitions [132]. To determine if micronutrient intake is associated with an asthma severity, Smith *et al.* administered the block food frequency questionnaire to participants in a randomized clinical trial of asthmatics. The study included 1033 participants, aged 12–75. This investigation found out that genistein, a soy isoflavone, was the only nutrient that had a consistent association with asthma severity. None of the other nutrients evaluated were related to asthma rate when adjusted for known confounders. Increasing consumption of genistein was associated with better lung function in patients with asthma [133].

**Figure 2:**
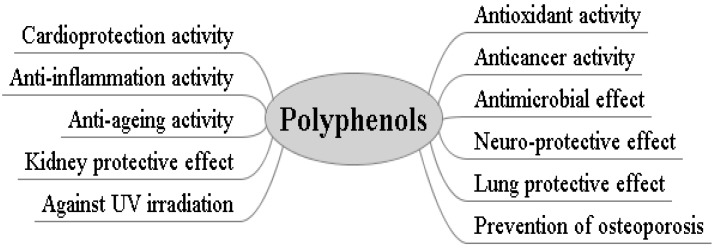
Some bioactivities of natural polyphenols.

Insulin resistance, a hallmark of metabolic disorders, is a risk factor for diabetes and cardiovascular disease. Concordant results were reported that polyphenols, such as resveratrol and epigallocatechin-3-gallate,* in vivo* had a beneficial effect on energy metabolism in diseases such as diet-induced obesity and insulin resistance [134,135]. Male C57BL/6J mice were fed either a normal chow diet or high-fat diet with or without epigallocatechin gallate supplement (50 mg/kg a day) for 10 weeks. Mice fed a high-fat diet with epigallocatechin gallate supplement gained less body weight and showed improved insulin sensitivity. In vehicle-treated high-fat diet mice, endothelial function was impaired in response to insulin but not to acetylcholine, whereas the epigallocatechin gallate-treated high-fat diet group showed improved insulin-stimulated vasodilation. Interestingly, epigallocatechin gallate intake reduced macrophage infiltration into aortic tissues in high-fat diet mice. The results showed that supplementation of epigallocatechin gallate improved glucose tolerance, insulin sensitivity, and endothelial function [136]. In addition, the morbidity of osteoporosis could be reduced by consumption of polyphenols. Eating more genistein, daidzein or their glycosides for several weeks could prevent the loss of bone mineral density and trabecular volume [1]. Forty-one 12 week-old female SD rats were assigned to five groups, and all rats were fed a low Ca (0.1%) diet *ad libitum*. Daily genistein dosage was 12 mg/kg body weight. The experimental duration consisted of the adaptation and treatment periods of 4 weeks each. The femoral bone mineral density (mg/cm^2^; mean ± SD), assessed by DEXA, of ovariectomized was significantly lowered to 206 ± 5 by −9%, when compared with 226 ± 2 of sham operated. The bone mineral density of ovariectomized-received genistein, ovariectomized-exercised and ovariectomized-received genistein-exercised were 217 ± 2, 217 ± 2 and 222 ± 2, respectively. Genistein dosage and exercise equally increased the bone mineral density of ovariectomized rats by 5%. Combined treatment of genistein and exercise more successfully recovered their decreased bone mineral density by 8%. Bone mineral density of the fourth lumbar vertebrae in ovariectomized was declined to 191 ± 7 by −15%, as compared to 225 ± 4 in sham operated. Ovariectomized-exercised and ovariectomized-received genistein-exercised gained the bone mineral density by 6% to 205 ± 4 and 203 ± 3, respectively. These results suggested the combined treatment of genistein dosage and resistance exercise had more beneficial effects than their separate trials on the prevention of ovariectomized-induced bone loss [137]. All the bioactivities of polyphenols, mentioned above, are summarized into Figure 2.

## 4. Bioavailability

Because polyphenols provided the greatest effectiveness in disease prevention, it is essential to know their bioavailability. The common definition of bioavailability was the proportion of the nutrient that is digested, absorbed and metabolized through normal pathways [138]. The increase of lipid-bound polyphenols in serum could be detected, and as a result of the bioactivity of polyphenols. After a 48 h washout period with an antioxidant-poor diet, 12 fasting subjects (6 men and 6 women) consumed a single dose (400 mL) of a phenolic-rich juice. A slight increase was observed in serum lipid-bound polyphenols, associated with a significant decrease in serum lipid peroxidation products, from 2 to 6 h. Urinary excretion of phenolics increased and peaked at 2 h after the intake and persisted up to 6 h post intake [41]. Moreover, after two weeks of daily red wine consumption, plasma levels of total phenolic concentrations increased significantly. The trace levels of metabolites were detected in plasma, which could not be found in the control group [139].

It was found that polyphenols have affinities with some proteins after absorbed. *In vivo*, the complexation of polyphenol and β-glucan/protein affected the bioavailability and beneficial properties of both the individual components. For example, polyphenol-protein complex formation can affect the digestion ability of several digestive enzymes present in human’s body; similarly, it can also reduce or enhance the antioxidant activity of polyphenols [140,141]. Laurent *et al*. found about 43.9% of catechin, 85.3% of epicatechin and all dimers disappeared at the end of 2 h of intestinal incubation* in vitro* digestion model [142]. At the same time, some cells enzyme activities were decreased, such as alkaline phosphatase and sucrase-isomaltase aminopeptidase N. These results showed that polyphenols had interacted with pancreatic proteins, which were detected by unmasked from acetonitrile extraction.

Polyphenols are decomposed in digestion lumen by pH changes. The oligomers were less stable than the monomers at both acidic and alkaline pH. At simulated gastric juice (pH 1.8), decomposition of high polymerized oligomers of procyanidins might occur and the slight increase in dimers procyanidins was observed through gastric step. After 2 h of *in vitro* incubation, monomers and dimers were quite stable at pH 7 in intestine medium, but 20% dimers were degraded at pH 7.4, and all dimers disappeared in pH 8.5. At pH 7.5 with incubation for 2 h, 15%–34% of epicatechin was degraded, while catechin was stable [143]. In another study, the contents of the large intestine of pigs were used to model anthocyanin metabolism because pig and human intestinal microflora are similar. An anthocyanin extract was incubated anaerobically in the contents of the large intestine of freshly slaughtered pigs for 0, 0.5, and 6 h. After 6 h, anthocyanins were no longer detected, but three metabolites were identified as 3-*O*-methylgallic acid, syringic acid and 2,4,6-trihydroxybenzaldehyde. Results from this study suggested that consumption of anthocyanins could lead to the formation of specific metabolites in the human gut. These metabolites offered the protective effect against colon cancer might attribute to anthocyanin consumption [144].

The metabolism of several polyphenols was well understood. The aglycones could be absorbed from the small intestine. However, most polyphenols were presented in food in the form of esters, glycosides, or polymers that cannot be absorbed in their native form [138]. In a word, the forms reaching the blood and tissues are different from those present in food. It is very difficult but necessary to identify all the metabolites and to evaluate their bioavailability [145,146].

## 5. Potential Toxicity

In recent years, the potential toxicity of some polyphenols was reported, such as catechin to damage DNA in mice spleen cells. A noticeable DNA damage was induced in mice spleen cells disposed by higher concentration of catechin [147]. Moreover, grape extracts were found to promote mitomycin C inducing sister chromatid exchange at the concentration from 75 to 300 μg/mL in human peripheral blood lymphocytes [148]. At the same concentrations, the situation of mitomycin C-induced clastogenicity was enhanced by a mixture of caffeic acid, gallic acid and rutin hydrate. In addition, notably negative effects were observed in fibroblast and keratinocyte cell lines after exposure to high concentration of epicatechin for 24 h or more time. Furthermore, the compounds with a gallate group exhibited more potential toxicity than those without the gallate group [149]. The results indicated that positive effects could be obtained from polyphenols in a safe concentration range. However, the concentrations of polyphenols were not the only crucial factor; negative effects of polyphenols were related to the synergistic effect and exposure time. Therefore, the dose and composition of polyphenols should be investigated further for secure and healthy application [17].

## 6. Conclusions and Prospects

This review provided a current understanding on the bioactivities of natural polyphenols and the benefits to human health. Over the last 20 years, polyphenols have been studied for their potential involvement in many areas including cancer, cardiovascular problems, inflammation and microbial diseases. Their protective activity was firstly attributed to their antioxidant, free radical scavenger, and metal chelator properties, then to their ability to inhibit or reduce different enzymes. The emerging findings suggested the interaction with signal transduction pathways and cell receptors had an effect on polyphenols’ biological activity. The achievable concentrations of polyphenols in the circulation after ingestion as well as the possibility of conjugation and metabolism of polyphenols were not illuminated very clearly. The exposure to high concentration of polyphenols, and over a long period, could induce DNA damage and obtain notably negative effects. Large scale randomized clinical trials should be conducted before the therapeutic use of polyphenols against human diseases can be fully established. The role of polyphenols in human health is still a fertile area of research. Future research should be focused on the deep mechanism *in vivo*, bioavailability of polyphenols, interaction between polyphenols, and so on.
